# Is Resource Allocation that is Sensitive to Vaccination Status Coercive? Who Cares?

**DOI:** 10.1080/15265161.2024.2353808

**Published:** 2024-06-24

**Authors:** Tess Johnson

**Affiliations:** University of Oxford

Park and Davies (2024) discuss the allocation of scarce resources during a pandemic—primarily ventilators and healthcare staff—and assess the ethical concepts that are used in arguments for and against using vaccination status as an allocation criterion. Whilst they aim to provide a “comprehensive framework for thinking about healthcare consumption in pandemic scarcity” (Park and Davies 2024, 67), the authors make a notable conceptual omission: they do not consider the potential result of vaccination status-sensitive resource allocation in coercing individuals to get vaccinated among the morally relevant considerations they list, which include responsibility, reciprocity, and justice. This omission is notable because of its salience in public and academic discussion during the COVID-19 pandemic (Nuffield Council on Bioethics 2020). The authors’ neglect of coercion, then, might be seen as a grave lapse for a “comprehensive” framework. In this commentary, I argue that the omission is quite right: if allocating healthcare resources on this basis is coercive, who cares?

I proceed as follows: after outlining relevant parts of Park and Davies’ work, I explain how coercion might be seen as a morally relevant factor in determining whether resource allocation should be vaccination status-sensitive or not. I then discuss how coercion in fact should be considered a descriptive concept—that is, whilst it may be a characteristic of certain actions, it is not morally valent, but merely a sometimes-proxy for other, properly moral factors, such as those discussed by Park and Davies. I conclude that we should not care whether vaccination status-sensitive resource allocation ends up coercing people to get vaccinated; rather, we should care whether people should get vaccinated, and whether there are good reasons for them not to get vaccinated.

Park and Davies focus on three ethical concepts that can be used to argue for or against vaccination status-sensitive resource allocation under conditions of pandemic scarcity.

Regarding responsibility, they discuss the idea that “patients should sometimes be held responsible for their health-affecting choices” (Park and Davies 2024, 67). To hold someone morally responsible, two conditions must be met: first, that their choice whether to get vaccinated is foreseeably connected to increasing healthcare resource scarcity; second, that their choice be “factually tractable” (2024, 68), or that the information on the choice is easily verifiable and accessible. They briefly connect the question of responsibility to whether this deserves retribution through reduced healthcare entitlements, but do not consider how the threat of reduced entitlements might change behaviors surrounding vaccination.

Regarding reciprocity, Park and Davies hold that in order for someone to have failed an obligation of reciprocity with regard to protecting the healthcare system through getting vaccinated, two requirements again pertain. These include fitness (where the recipient of a good such as health protection through a well-resourced healthcare system perceives it as a good) and proportionality (where a recipient gets at least as much out of healthcare access as they put in through vaccination). They note that even if a failure of reciprocity is established, further argument is required to justify giving vaccine-refusers lower treatment priority.

Finally, regarding justice, the authors discuss how unjust background conditions intersect with the two above factors, meaning that judging failures of responsibility and of reciprocity must be tempered according to the conditions that lead to some having reasonable opportunities to get vaccinated or not. They hold that barriers to vaccination access and trust of healthcare workers must be taken into account particularly by those wishing to argue for vaccination status-sensitive resource allocation.

During the COVID-19 pandemic, these three concepts (responsibility, reciprocity, and justice) were certainly not the only ones underlying arguments for and against vaccination-sensitive measures. Vaccine passports for international travel and for access to public venues, in particular, were talked about in terms of their potential violation of a right to privacy (Ortiz-Millán 2023), their political legitimacy (Barnhill, Bonotti, and Susser 2023) and whether they would coerce people into being vaccinated (Nuffield Council on Bioethics 2020). Why shouldn’t Park and Davies focus on the potential for coercion in their discussion of vaccination status-sensitive resource allocation, too?

The answer I argue for is that whilst coercion seems to be a prominent public concern when it comes to pandemic responses, and particularly to vaccination, it is not in itself morally valent. The idea that allocating ventilators on the basis of whether prospective patients have been vaccinated could induce people to get vaccinated against their wills should not be used as an argument against it. That is not to say that vaccination status-sensitive resource allocation could not count as coercion. Indeed, it would according to canonical definitions including Nozick’s, based on threat of negative consequences (1969), and accounts centering on the enforcement of actions through exploiting power dynamics (Anderson 2010). But that coercion need not be wrong. Anderson’s account is open to both morally valent and neutral conceptions of coercion, based on whether the exploitation of power dynamics for enforcement is considered legitimate (such as in relationships between state and citizens or parent and child) or illegitimate (such as in relationships between those of equal moral status, e.g., political or social groups). Nozick’s account is more often conceived as moralized, based on drawing an equivalence between the threat to impose negative consequences on another and harming another. Whilst coercion can still be justified according to some moralized accounts, it is a *pro tanto* wrong which must be outweighed by reasons in favor of coercion (such as its maximizing utility, promoting justice, etc.).

I argue that coercion is more appropriately considered a descriptive, rather than moral, concept: whether a coercive action is right or wrong does not depend on its coerciveness. After all, we accept coercion in many parts of our lives, from parents preventing their children from climbing trees to the prevention of secondhand smoking harms through state regulation. Even in cases where coercion sits in the context of illegitimate power dynamics between actors, that may be a matter of wrongful power: coercion acts as a proxy for the actual wrongness, which is in the existence of power differentials between two groups of moral equals. I hold that the label of coercion often obscures properly moral determinants of whether a coercive action is right or wrong, as in cases when a coercive action undermines justice or is less effective at achieving valuable goals than alternatives. These properly moral determinants might be based on consequentialist or teleological reasoning such as whether the action promotes utility or virtue, or on principlist grounds such as whether it is universalizable or based on good intention.

What matters, then, is whether an action is justified according to concepts like those Park and Davies explore, including responsibility, reciprocity, and justice. I have argued elsewhere that coercive public health measures can be justified through the establishment of responsibility and the legitimacy of intervention (Johnson and Matlock 2023), or through the prevention of harm to others (Johnson 2024a), or through the promotion of justice, establishment of a duty of easy rescue, or paternalism (Johnson 2024b). These arguments apply particularly well in decision-making surrounding potentially coercive antimicrobial stewardship policies. For instance, insofar as we can hold that livestock farmers in high-income countries have a collective duty of easy rescue to protect the antimicrobial commons by not feeding human-critical antibiotics to healthy livestock, stricter regulation of access to antibiotics may be a good choice of state action (Johnson 2024a). Notably, whether the action is right or wrong also depends on contextual factors that determine whether the requirements of each moral argument is fulfilled. Similarly to Park and Davies’ highlighting the role of unjust background conditions, I emphasize the need for context-specificity in judgements of coercive action in the name of public health. The coercive results that may be associated with vaccination status-sensitive resource allocation are not so different. Whilst any public health action requires justification, what is needed is attention to whether the requirements (both value-based and empirical) of arguments to justify the action are fulfilled. The role of coercion in such measures is simply as a potential effectiveness amplifier: coercion may better promote any ends, whether good or bad.

Park and Davies highlight how, under certain circumstances, arguments for (and against) vaccination status-sensitive resource allocation might be based on the establishment of responsibility for getting vaccinated or the failure to uphold a reciprocity-based obligation to be vaccinated. They have noted how these arguments depend on circumstances including whether background conditions are just. I agree with their approach and want to commend them on not falling prey to salience over substance: they have omitted to consider the possible coercive effects of resource allocation on the basis of vaccination status, and are right to do so. Coercion is not morally valent, but rather a sometimes-proxy for actual ethical arguments. If the question is, ‘What about coercion?’ the appropriate answer is, ‘Who cares?’
